# Global experiences in health workforce policy, planning and management using the Workload Indicators of Staffing Need (WISN) method, and way forward

**DOI:** 10.1186/s12960-021-00695-9

**Published:** 2022-01-28

**Authors:** Teena Kunjumen, Mollent Okech, Khassoum Diallo, Pamela Mcquide, Tomas Zapata, James Campbell

**Affiliations:** World Health Organisation, Geneva, Switzerland

Health systems can only function with health workers; improving health service coverage, and realizing the right to the enjoyment of the highest attainable standard of health is dependent on their availability, accessibility, acceptability and quality of service [1]. When health workers are equitably distributed and accessible by the population, and possess required competencies, they are motivated and empowered to deliver quality care that is appropriate and acceptable. The World Health Organization (WHO) strives to build country capacity to plan for health workforce using data for evidence-based informed policies and decisions. WHO Global Strategy on Human Resources for Health (GSHRH) 2030, adopted by WHA 69.16 [2], aims to build the capacity of institutions at subnational, national, regional and global levels for effective public policy stewardship, leadership and governance of actions on human resources for health. It outlines the need for strengthening data on HRH for monitoring and ensuring accountability for the implementation of health plans and strategies, through the progressive implementation of the National Health Workforce Accounts (NHWA) [3]. The UN Sustainable Development Goals also aim to improve the health workforce situation in countries [4]. 2021, the Year of Health and Care Workers drew further attention to health care workers, largely due to the ongoing COVID-19 pandemic [5]. The foundation for a strong and effective health workforce, able to respond to the changing health needs, requires mapping the supply and skills of health workers to population needs now and for the future through evidence-based planning.

Countries that have been planning for health workforce using simple population ratios and health worker density benchmarks, realize that these traditional methods on their own no longer support the complexity of population health needs or emergency situations. Health policy planners need to be equipped to make decisions on the recruitment and deployment of health workers at the primary, secondary and tertiary-level health facilities, considering the evolving health delivery models and population demands. A recent study on the impact of COVID-19 on health workforce argues for the need for periodic and systematic data recording and analysis to enable efficient health workforce planning for effective service delivery [6]. Effective and efficient planning can only be achieved with supportive data and evidence. The WHO Workload Indicators of Staffing Need (WISN) tool serves this very purpose [7]. Unlike many health workforce planning tools that consider available head count only, the WISN methodology uses service data and actual available working time (AWT) of health workers to execute both, health related and administrative related tasks [7]. This provides the evidence needed to plan and deploy the required health workforce, with required skill-mix in the required facilities. WISN methodology also emphasizes the principles of multi-stakeholder governance and engagement through Steering Committee, Technical Task Force and the Expert Working groups as outlined in the WISN user manual [7].

Many countries have implemented WISN since WHO launched the revised WISN manual and the automated multilingual WISN software tool, in 2010. The growing online WISN user’s community represents users from over 140 countries with close to 1,500 members that share WISN experiences and opportunities [8]. The WISN tool has been able to cater to varying needs and generate the evidence required to make policy decisions regarding adequate staffing, policies for role distribution, revising national staffing norms, upgrading health facilities, determining staffing requirements for specific health interventions, and prioritizing staffing needs and skill-mix. However, the lessons, experiences and the challenges of the implementation and the governance as well as the methodological aspects have not been documented enough. At the first ever global WISN experts meeting in Istanbul, 2019, it was agreed that WHO organise efforts to bring together a global body of evidence from around the world.

Supporting the GSHRH bid to evidence-informed planning and capacity-building, this supplementary issue ‘*Countries’ experiences on implementing WISN methodology for health workforce planning* documents experiences from countries and aims to inspire and inform more verifiable actions with evidence to empower health planners and policy-makers. WISN studies and their results influence national health workforce planning, estimate health worker requirements for different settings, enable equitable distribution of the health workforce, develop or revise staffing norms, and improve information systems. The body of evidence in the 17 articles strongly suggests that WISN, is adaptable to varying national contexts and complements other existing planning tools to achieve a broader purpose. The articles demonstrate that WISN can be applied at primary through to tertiary-level facilities, in emergency settings, and all health occupations.

Primary care health facilities serve as the cornerstone for building a strong healthcare system that ensures positive health outcomes and health equity [9]. Increasing number of countries are shifting their focus from disease-oriented programmes to person/family-focused and community-oriented primary care services considering the cultural and environmental influences. For efficient and effective health service delivery, it is critical to have adequate staffing at all health care levels. Al-Dabbagh et al. [10], Okoroafor et al. [11], Aytona et al. [12], Bonfim et al. [13], Thu et al. [14], Menezes et al. [15], Nair et al. [16] present studies from Iraq, Nigeria, Philippines, Brazil, Vietnam and India, demonstrating how WISN methodology can be used to determine staffing requirements at all levels including primary care facilities and tertiary hospitals.

Most efforts in many countries focus on “traditional” health worker occupations (e.g., doctors and nurses), ignoring a range of other professions integral to providing quality health services and addressing the social determinants of health. Stankovic et al. [17], Machado et al. [18], Menezes et al. [15], Silva et al. [19] conducted staffing requirements for bio-chemical laboratory staff, orthopedists, palliative care teams, gynecologists and obstetricians, to understand effective skill-mix in health service teams. NHWA implementation and its annual reporting provides an opportunity to acknowledge all occupations and thus many countries are beginning to use WISN to determine the skill-mix required for the different health teams.

WISN studies equip health workforce policy-makers with information to define national standards, revise staffing norms, improve staff distribution and reclassify health facilities. The experiences from Bangladesh, Oman, Philippines and Papua New Guinea are presented by Nuruzzaman et al. [20], Elfaki et al. [21], Dimiri et al. [22] andAytona et al. [12]. The multi-country study on planning reproductive maternal newborn child health (RMNCH) services in Bangladesh, Ghana, Kenya, Oman and Papua New Guinea by Kunjumen et al. demonstrates that evidence-based workforce planning must be context-specific requiring that each country develop its own workload components and activity standards aligned to their local contexts and that activity standards cannot be adopted or adapted from one country to another despite having similar workload components [23]

Knowing your health staff requirements for palliative care, trauma care and in emergency situations play a huge role in ensuring timely and efficient care. While Silva et al. demonstrates how WISN can be used to assess staffing need in a palliative care team [19], Haroon et al. used the WISN methodology to identify staff gaps in Pakhtunkhwa Province of Pakistan that would hamper an adequate response to public health emergencies [24]. On the other hand, McQuide et al., utilized the service standards set up in WISN studies and another WHO tool—The Health Workforce Estimator (HWFE) [25] to estimate the required number in each relevant health occupation to determine staffing requirements for COVID-19 management in Mali and Kenya [26]. The results demonstrate that the WISN approach applied to the Health Workforce Estimator tools can be readily adapted to the local context to rapidly estimate the number of health workers and beds needed to respond to the predicted COVID-19 pandemic caseload.

To conduct a WISN study, one needs to master the methodology outlined in the WISN User manual which includes the scientific calculations and implementation mechanism built on the intended scope of the study [7]. The study by Namaganda documents lessons learned from a Delphi study that rated eight technical steps of WISN [27]. Key lessons learned were that the benefits gained from applying the WISN methodology outweighs the challenges faced in understanding the technical steps and that starting with small-scale projects sets the ground for more effective scale-up than attempting massive national application of the methodology for the first time, amongst others. This translates well to the agreement at the global experts meeting in Istanbul, that the selected sections of the WISN user manual and user interface features of the WISN software need to be enriched to guide users to relevant policy and management decisions. While the WISN methodology is straightforward to implement using primarily micro-data from health facilities, it is conceded that analysis using macro-level data is equally required to strengthen health workforce projection and forecasting.

In conclusion, the body of evidence shows that WISN continues to be a relevant and effective tool for health workforce planning which when complemented with other tools can provide a comprehensive outlook to support policy and planning dialogue, decision-making and investment in health [28]. In the post-COVID-19 economy, where countries face challenges in funding the health and social sector, the need for efficient health workforce planning to attain optimal productivity and performance, cannot be further emphasized. The complementarity of WISN implementation and NHWA implementation in countries, lies within the foundational similarities of multi-stakeholder engagement and the use of multiple data sources to build comprehensive health information systems that are multi-sectoral. It should be noted that both, NHWA and WISN implementation, improve data availability for workforce planning. Globally, countries lack comprehensive data on human resources for health needed for an in-depth Health Labour Market Analysis (HLMA) to guide national health workforce strategies, and empower policy-makers and health workforce planners to take better decisions [29]. Therefore, it is crucial to build on the NHWA implementation, WISN implementation, and HLMA studies to strengthen the capacities of countries. This will progressively improve health workforce information for better policy decision-making and contribute to the objectives of the WHO Global Strategy on Human Resources for Health: Workforce 2030.

## Abbreviations

AWT: Available Working Time
COVID-19: CoronaVirus Disease 2019
GSHRH: Global Strategy on Human Resources for Health
HWFE: Health Workforce Estimator
HLMA: Health Labour Market Analysis
NHWA: National Health Workforce Accounts
WISN: Workload Indicators of Staffing Need
WHA: World Health Assembly
WHO: World Health Organization
